# Exploration of Metabolite Biomarkers to Predict the Efficacy of Dupilumab Treatment for Atopic Dermatitis

**DOI:** 10.1155/2023/9013756

**Published:** 2023-11-01

**Authors:** Shoko Miyamoto, Yasutomo Imai, Masako Matsutani, Makoto Nagai, Kiyofumi Yamanishi, Nobuo Kanazawa, Shin Nishiumi

**Affiliations:** ^1^Department of Dermatology, Hyogo Medical University, 1-1, Mukogawa-cho, Nishinomiya, Hyogo 663-8501, Japan; ^2^Department of Dermatology Institute of Biomedical Sciences, Tokushima University Graduate School, 2-50-1, Kuramoto-cho, Tokushima, Tokushima 770-8503, Japan; ^3^Imai Adult and Pediatric Dermatology Clinic, 5-1-1, Ebie, Fukushima-ku, Osaka, Osaka 553-0001, Japan; ^4^Department of Dermatology, Mie University Graduate School of Medicine, 2-174, Edobashi, Tsu, Mie 514-8507, Japan; ^5^Department of Omics Medicine, Hyogo Medical University, 1-1, Mukogawa-cho, Nishinomiya, Hyogo 663-8501, Japan; ^6^Department of Biosphere Sciences, School of Human Sciences, Kobe College, 4-1, Okadayama, Nishinomiya, Hyogo 662-8505, Japan

## Abstract

Dupilumab (DUP) is the first biological agent used treating atopic dermatitis (AD). Notwithstanding its high cost, the type of patient group for which the drug is effective remains unclear. In this retrospective study, we aimed to identify novel and reliable biomarkers which can be measured before DUP administration and to predict the efficacy of DUP. Serum samples from 19 patients with AD treated with DUP were analysed by metabolome analysis using gas chromatography–mass spectrometry. Total 148 metabolites were detected, and the relative values of the metabolites were compared between the patient group that achieved 75% improvement in Eczema Area and Severity Index 16 weeks after administration of DUP (high responders: HR; *n* = 11) and that did not (low responders: LR; *n* = 8). The HR and LR groups had significant differences in the relative values of the eight metabolites (lactic acid, alanine, glyceric acid, fumaric acid, nonanoic acid, ribose, sorbitol, and ornithine), with ribose emerging as the best. Furthermore, we evaluated the serum concentrations of ribose and found that ribose may be a useful metabolite biomarker for predicting the efficacy of DUP in AD.

## 1. Introduction

Atopic dermatitis (AD) is characterised by an itchy eczema with recurrent exacerbations and relapses. The Eczema Area and Severity Index (EASI) score is used to measure the severity of AD, with higher scores reflecting worse severity. EASI-75 responders are participants who have achieved 75% improvement in EASI score from the baseline. Dupilumab (DUP) is a human monoclonal antibody that blocks the signalling of interleukin-4 and interleukin-13 and has been used for the treatment of AD in Japan from April 2018. Although DUP treatment is effective in several patients with AD in the clinical practice, a few do not respond adequately to it. In the CHRONOS trial [1], 69% patients achieved EASI-75 16 weeks after DUP administration; however, the remaining 31% did not, and the factors predicting the efficacy of DUP were unknown [1]. Another clinical trial also reported that DUP had similar effects regardless of eosinophil count, thymus and activation-regulated chemokine (TARC) and IgE at baseline [2], and there are currently no reliable predictors. The high cost of DUP treatment is a major concern in the choice of treatment for AD because patients typically pay 30% of their medical expenses in Japan. Therefore, identifying robust biomarkers to predict the efficacy of DUP prior to treatment is essential. Recently, metabolome analysis has been attracting attention and applied to the search for biomarkers for diagnosis and treatment, especially in the field of cancer. However, there have been few studies using metabolome analysis in the field of dermatology. In this retrospective study, we aimed to identify the biomarkers that predict the efficacy of DUP in patients with AD using metabolome analysis.

## 2. Materials and Methods

### 2.1. Study Participants and Data Collection

This study included 19 male patients with typical features of AD out of 109 patients with AD who commenced DUP at our hospital between April 2018 and July 2020. Patients with prurigo-type AD, associated with predominantly excoriated nodular lichenified lesions [3], were excluded because the surface area of the skin rash was small and the EASI score cannot be accurately measured. This study complied with the guidelines of the Declaration of Helsinki and was approved by the Ethics Committee of Hyogo Medical University (permission number 0212). Written informed consent was obtained from all the participants prior to their enrolment in the study. The patients satisfied all the following criteria: (1) they were under appropriate treatment, with topical steroids (strong class or higher) or topical calcineurin inhibitors at least for the last 6 months, (2) Investigator's Global Assessment score was ≥3, (3) EASI score was ≥16 or EASI score of head and neck was ≥2.4, (4) body surface area score was ≥10%. The patients received 600 mg of DUP as a loading dose and 300 mg every 2 weeks thereafter, in addition to topical steroids or calcineurin inhibitors during DUP treatment. The topical applications and emollients were used according to the proactive therapy recommended by the Japanese AD guidelines [4]. Clinical and laboratory data were collected at baseline (before DUP treatment). Disease severity was determined using the EASI and Dermatology Life Quality Index (DLQI) at baseline and 16 weeks after DUP administration. Serum samples for metabolome analysis were obtained in the morning under fasting conditions at baseline and 16 weeks. In this study, the primary goal of AD treatment was to achieve EASI-75. We defined patients who achieved and did not achieve EASI-75 at 16 weeks as high responders (HR) and low responders (LR), respectively. In addition, we defined patients who achieved and did not achieve EASI-90 at 16 weeks as super high responders (SHR) and non-super-high responders (non-SHR), respectively. A secondary goal of AD treatment was to achieve a 4-point or greater improvement in the DLQI from baseline. Patients who achieved 4-point or greater improvement at 16 weeks were defined as DLQI-high responders (DLQI-HR) and those who did not as DLQI-low responders (DLQI-LR).

## 3. Materials

2-Isopropylmalic acid (2-IPMA), methoxyamine hydrochloride, and D-ribose were purchased from Sigma–Aldrich Japan (Tokyo, Japan). N-Methyl-N-trimethylsilyltrifluoroacetamide (MSTFA) was purchased from GL Sciences, Inc. (Tokyo, Japan). A standard alkane series mixture (C7–C33) was obtained from Restek Co. (PA, USA). [U-^13^C_5_]-labelled D-ribose was purchased from Cambridge Isotope Laboratories, Inc. (Andover, MA, USA).

### 3.1. Pretreatment for Gas Chromatography–Mass Spectrometry (GC/MS)-Based Metabolome Analysis

For extraction of metabolites from the serum, 50 *µ*L of each sample was mixed with 250 *µ*L of methanol and 10 *µ*L of 0.5 mg/mL 2-IPMA, and then shaken at 1,200 rpm for 30 min at 37°C before being centrifuged at 20,600 × g for 5 min at 4°C. Next, 225 *µ*L of the supernatant obtained from each mixture was added to a clean tube, centrifuged, and freeze-dried overnight. For oximation, 60 *µ*L of 20 mg/mL methoxyamine hydrochloride dissolved in pyridine was added into the tube, and then sonicated for 20 min before being shaken at 1,200 rpm for 90 min at 30°C. The mixture was centrifuged at 20,600 × *g* for 5 min at 20°C, and then 40 *µ*L of the resultant supernatant was subjected to GC/MS as described in the “GC/MS analysis” section.

### 3.2. GC/MS Analysis

GC/MS analysis was performed using an AOC-6000 (Shimadzu Co., Kyoto, Japan) and GCMS-TQ8040 (Shimadzu Co.) equipped with a BPX-5 capillary column (internal diameter: 30 m × 0.25 mm; film thickness: 0.25 *µ*m; SEG, Victoria, Australia). In the AOC-6000, 20 *µ*L of MSTFA were added to the sample supernatant, and then the mixture was incubated at 750 rpm for 30 min at 37°C before 1.0 *µ*L of the derivatised solution was injected into the GCMS-TQ8040. GC/MS analysis was performed using the Smart Metabolites Database Ver.2 (Shimadzu, Co.), which contains information regarding the GC analytical conditions, multiple reaction monitoring (MRM) parameters, and the retention index employed for metabolite measurement. To correct the retention time, the Automatic Adjustment of Retention Time (AART) function of the GCMSsolution software (Shimadzu Co.) and a standard alkane series mixture (C7–C33) were used. Peak identification was performed automatically and confirmed manually based on the specific precursor and productions, and the retention time.

### 3.3. Quantitative Analysis of D-Ribose

D-ribose was quantitatively analysed using the internal standard method with D-ribose and the corresponding stable isotope, [U-^13^C_5_]-labelled D-ribose. In brief, 50 *µ*L of each serum sample was mixed with 270 *µ*L of methanol containing [U-^13^C_5_] -labelled D-ribose. As pre-treatment for the GC/MS analysis, the mixture was treated according to the protocols described in the “Pretreatment for GC/MS-based metabolome analysis” section. The analysis was performed as described in the “GC/MS analysis” section. The serum concertation (*µ*M) of D-ribose was calculated based on the multipoint calibration curves derived from the peak area (*y*-axis; native/isotope) and concentration (*x*-axis; native/isotope) ratios.

### 3.4. Statistical Analysis

The normality of the data distribution was verified using Shapiro–Wilk test. Student's *t*-test and Mann–Whitney *U* test were used to compare means for normally and not-normally distributed values, respectively. Student's *t*-test was used to compare the relative values of metabolites at baseline analysed using metabolome analysis. Based on the metabolites that exhibited significant differences, principal component analysis (PCA) was performed to differentiate between the HR and LR samples. For the metabolites displaying significant differences, logistic regression and receiver operating characteristic (ROC) analyses were performed to estimate the area under the curve (AUC). The serum concentration of the metabolite with the highest AUC was then provided for logistic regression and ROC curve analyses to estimate its performance as a predictive biomarker. The optimal cut-off value was determined using the Youden index. Changes in the EASI at 16 weeks from baseline were calculated as percentages. The change in EASI was compared based on the predicted probability using Student's *t*-test. The relative values of the metabolites at baseline were also compared between the SHR and non-SHR groups and between the DLQI-HR and DLQI-LR groups using Student's *t*-test and Mann–Whitney *U* test. Changes in the relative values of metabolites at 16 weeks from baseline were calculated as percentages. Spearman's *R* correlation was used to measure the association between changes in the EASI and relative values of the metabolites. Statistics were calculated using GraphPad Prism 9 and JMP Pro 16.

## 4. Results

**Figure 1:**
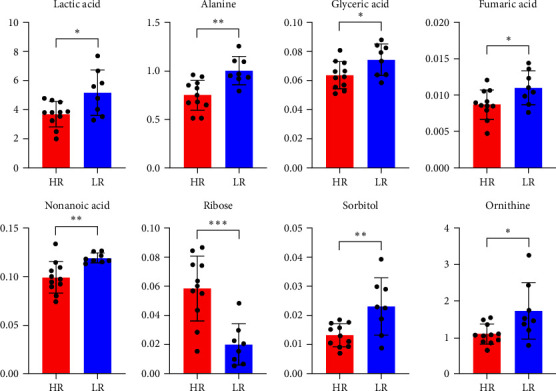
Relative values of the eight metabolites at baseline in HR and LR patients. The *x* and *y* axes represent the patient groups and relative values of each metabolite, respectively. The normality of the data distribution was verified using Shapiro–Wilk test. Statistical significances were analysed using Student's *t*-test. Error bar is standard deviation;  ^*∗*^*p* < 0.05,  ^*∗∗*^*p* < 0.01, and  ^*∗∗∗*^*p* < 0.001.

**Figure 2:**
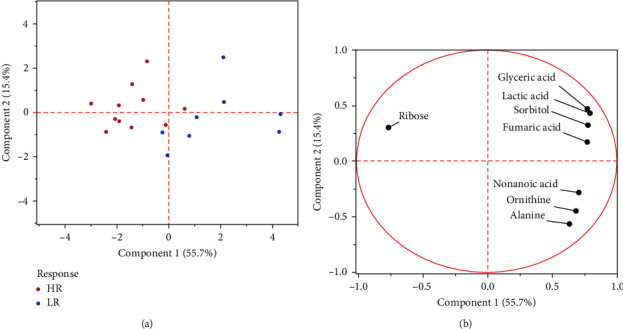
Comparative principal component analysis of the HR and LR groups based on the relative values of the eight metabolites: (a) Score plot and (b) Loading plot.

**Figure 3:**
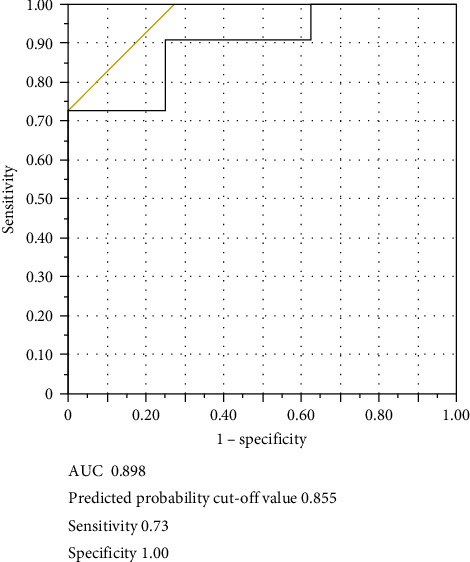
ROC curve for the serum concentration of ribose. The logistic regression and ROC analyses were performed using the serum concentration of ribose. Optimal cut-off value was determined using the Youden index. ROC: receiver operating characteristic; AUC: area under the curve.

**Figure 4:**
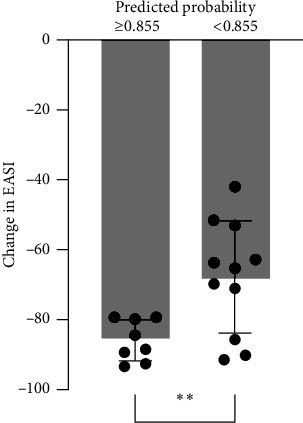
Change in EASI at 16 weeks from baseline between patient groups with predicted probabilities ≥0.855 and <0.855. The optimal predicted probability cut-off value of 0.855 was determined in Figure 3. The *x*-axis represents the patient groups with predicted probabilities ≥0.855 and <0.855; the *y*-axis represents the percent change in EASI at 16 weeks from baseline. The normality of the data distribution was verified using Shapiro–Wilk test. Statistical significances were analysed using Student's *t*-test. Error bar is standard deviation;  ^*∗*^*p* < 0.05,  ^*∗∗*^*p* < 0.01, and  ^*∗∗∗*^*p* < 0.001.

Table 1 presents the characteristics of the patients. Eleven and eight patients were included in the HR and LR groups, respectively. In total, 148 metabolites were detected in the serum samples using metabolome analysis. Among these, the relative values of eight metabolites (lactic acid, alanine, glyceric acid, fumaric acid, nonanoic acid, ribose, sorbitol, and ornithine) exhibited significant differences at baseline between the HR and LR groups by the Student's *t*-test (Table 2, Figure 1; *p*=0.0159, 0.0021, 0.0356, 0.0369, 0.0044, 0.0005, 0.0071,  and 0.0220, respectively). PCA revealed that HR and LR could be distinguished based on the relative values of the eight metabolites, and ribose was the metabolite that characterised HR (Figure 2). Logistic regression and ROC analyses were performed using the relative values of the eight metabolites. The AUC of ribose (0.920) was the highest among the eight metabolites (Table 3). To examine the results of ribose in greater detail, ribose was quantitatively analysed, and the calculated serum concentration was used for logistic regression and ROC analyses (Table 4). The AUC value was 0.898, and the predicted probability cut-off value of 0.855 in the ROC curve yielded a sensitivity of 73% and a specificity of 100% (Figure 3). There was a significant difference in the change of EASI at 16 weeks from baseline between patients with predicted probabilities of ≥0.855 and <0.855 by the Student's *t*-test (Figure 4; *p*=0.0081). Four and 15 patients were included in the SHR and non-SHR groups, respectively. Among the eight metabolites, the relative values of two metabolites (glyceric acid and fumaric acid, but not ribose; *p*=0.0203, 0.0145, 0.5955) displayed significant differences between the SHR and non-SHR groups by the Student's *t*-test and Mann–Whitney *U* test. Fifteen and four patients were included in the DLQI-HR and DLQI-LR groups, respectively. The relative values of all eight metabolites did not significantly differ between DLQI-HR and DLQI-LR groups by the Student's *t*-test and Mann–Whitney *U* test. All four patients in the DLQI-LR group were included in the HR group and had a relatively low-baseline DLQI of less than 7. No correlation was found between the change in EASI and the relative values of ribose at 16 weeks from baseline (*r* = −0.0525).

## 5. Discussion

In this study, eight metabolites exhibited significant differences between HR and LR groups before the administration of DUP, and logistic regression and ROC analyses suggested that ribose could be the best candidate biomarker. The serum concentration of ribose was measured to apply the results to the clinical practice, and logistic regression and ROC analyses were reperformed to evaluate its ability as a biomarker. The results indicate that ribose could be a useful biomarker for predicting the efficacy of DUP. The reason for the efficacy of DUP in patients with high levels of ribose is unclear; however, ribose is associated with poly (ADP) ribose polymerase (PARP), which is involved in numerous cellular processes such as DNA repair, protein turnover, inflammatory regulation, ageing, and metabolic regulation [5]. Additionally, PARP may play a critical role in AD [6, 7]. A few studies have reported the use of metabolomics or lipidomics for AD; however, ribose has not yet been discussed [8–14]. The values of ribose in healthy subjects have not been clearly defined in previous studies, and they were not measured in this study. Comparing ribose levels in healthy subjects and AD patients may help us understand the role of ribose in AD and is an important topic for future research. Using metabolomics and lipidomics, Zhang et al. [15] demonstrated that DUP treatment for patients with AD affected the concentrations of specific metabolites and altered specific metabolic pathways associated with them, including glycerophospholipid metabolism, valine, leucine and isoleucine biosynthesis, citrate cycle, arachidonic acid metabolism, pyrimidine metabolism, and sphingolipid metabolism. [15]. Ribose is not included in these metabolic pathways, but may be slightly related to pyrimidine metabolism because it is a nucleotide component. Further investigation into the relationship between ribose and the mechanism of DUP treatment for patients with AD is required.

Metabolome analysis, an omics technology, is the comprehensive study of low-molecular-weight metabolites in the body, including saccharides, amino acids, and fatty acids. Metabolome data cover information based on environmental factors as well as information derived from genetic factors because metabolites are the downstream products of cellular processes, including deoxyribonucleic acid, ribonucleic acid, and proteins. Therefore, the metabolome, which is closer to the phenotype, has been recognised as useful for the detailed evaluation of final phenotypes, and metabolome analysis has been widely applied in biomarker research [16].

Nettis et al. [17] considered complete responders as patients who achieved all three domains (EASI-75, peak pruritus Numerical Rating Scale (PP-NRS) ≥ 4 point improvement, and DLQI ≥ 4 point improvement). Predictors of complete responders after 16 weeks of DUP treatment were lower age, low baseline PP-NRS score, early onset AD, history of allergic conjunctivitis, and complete responders after 4 weeks of DUP treatment. Ariëns et al. [18] reported that the baseline EASI was significantly higher among patients who achieved all the previously described three domains compared with those who did not after 16 weeks of DUP treatment. Ferrucci et al. [19] reported that the predictors of patients who achieved EASI-75 after 4 weeks of treatment were early AD onset and the absence of hypereosinophilia (>500 × 10^3^/L), while no predictors achieved EASI-75 after 16 weeks of treatment. Linder et al. [20] defined patients who achieved a SCORing Atopic Dermatitis (SCORAD) score of 75 after 16 weeks as optimal responders, and their predictors were lower weight and less frequent exposure to tobacco and alcohol. Olesen et al. [21] found that the predictors of patients who achieved EASI-75 were female sex, lower age, lower baseline EASI, and lower baseline IgE after 4 weeks of treatment, and only female patients after 12 weeks. Lee et al. [22] found that the predictors for patients who received DUP treatment every 4 weeks and achieved EASI-75 after 16 weeks were low baseline eosinophil count and low baseline LDH level. Thus, although several researchers have reported factors predicting the efficacy of DUP, the results vary and consistent conclusions have not yet been reached.

The non-correlation between the changes in EASI and in the relative values of ribose suggests that ribose does not reflect disease activity during DUP treatment. Discrepancy was found between the changes in the EASI and DLQI in this study. The reason may be that patients with severe to moderate disease in this study had fewer subjective symptoms owing to chronic symptoms of AD, and a few in the HR group had a low-baseline DLQI.

This study has a few limitations. First, the number of patients was too small, and the biomarkers suggested in this study were exploratory. Second, this study was conducted using data from a single centre in Japan, and the results may differ according to race, ethnicity, or region. Third, only male patients were selected in this study to eliminate differences in metabolites between sexes. It is unclear whether similar results would be obtained in females.

## 6. Conclusions

Ribose may be a useful biomarker for predicting the effects of DUP. Our results provide clues for establishing personalised treatment regime for AD. Currently, several metabolites, including ribose, cannot be measured using routine blood tests. However, the means of measuring these metabolites will be established if their abilities as biomarkers are proven. The results of this study are exploratory, and a greater number of cases need to be investigated in the near future.

## Figures and Tables

**Table 1:** Characteristics of the patients.

Characteristics	Total (*n* = 19)
Age (years), mean (SD)	37.9 (12.0)
Sex, *n* (%)	
Male	19 (100)
EASI, mean (SD)	36.9 (15.1)
IGA, mean (SD)	3.3 (0.5)
POEM, mean (SD)	16.6 (7.0)
DLQI, mean (SD)	12.2 (7.3)
TARC (pg/mL), mean (SD)	4,256.5 (4013.3)
Eosinophil (/*µ*L), mean (SD)	773.9 (660.4)
LDH (IU/L), mean (SD)	323.9 (102.2)
IgE (IU/mL), mean (SD)	13,150.7 (25030.4)
Allergic comorbidity, *n* (%)	
Rhinitis	9 (47)
Asthma	3 (16)
Conjunctivitis	2 (11)

*Note*. SD: standard deviation; EASI: Eczema Area and Severity Index; IGA: Investigator's Global Assessment; POEM: Patient-Oriented Eczema Measure; DLQI: Dermatology Life Quality Index; TARC: thymus and activation-regulated chemokine.

**Table 2:** Change in EASI and relative values of the eight metabolites at baseline and 16 weeks in the 19 patients.

Patient	Change in EASI (%)	Lactic acid		Alanine		Glyceric acid		Fumaric acid		Nonanoic acid		Ribose		Sorbitol		Ornithine	
		Base line	16w	Base line	16w	Base line	16w	Base line	16w	Base line	16w	Base line	16w	Base line	16w	Base line	16w
1	−89	2.4889	2.9917	0.6746	0.9216	0.0664	0.0599	0.0079	0.0074	0.0927	0.1035	0.0572	0.1049	0.0159	0.0170	1.0434	1.3224
2	−93	3.8149	3.3289	0.8477	0.6685	0.0509	0.0565	0.0047	0.0055	0.0976	0.0848	0.0438	0.0568	0.0092	0.0086	0.8581	0.8845
3	−79	1.9904	4.0866	0.5142	0.9825	0.0570	0.0642	0.0090	0.0139	0.0746	0.0951	0.0553	0.0334	0.0112	0.0161	1.0689	1.8425
4	−89	4.6248	3.5115	0.6509	0.6761	0.0723	0.0678	0.0106	0.0067	0.0897	0.0831	0.0744	0.0619	0.0071	0.0084	1.0829	1.3529
5	−79	4.7877	3.5567	0.5143	0.6977	0.0808	0.0692	0.0105	0.0077	0.1063	0.1263	0.0750	0.0815	0.0153	0.0324	0.6537	1.3012
6	−94	3.8781	4.3780	0.9444	0.9464	0.0627	0.0672	0.0065	0.0053	0.0805	0.1092	0.0637	0.0084	0.0094	0.0115	1.3627	1.7566
7	−85	3.5302	3.7355	0.8714	0.9678	0.0555	0.0636	0.0086	0.0077	0.1095	0.1033	0.0847	0.0651	0.0131	0.0139	1.5498	1.6364
8	−80	3.8297	4.5453	0.9633	0.9685	0.0734	0.0709	0.0085	0.0063	0.1003	0.0997	0.0868	0.0146	0.0177	0.0195	0.9360	1.1365
9	−90	3.7840	4.9297	0.8221	1.2222	0.0619	0.0730	0.0085	0.0096	0.1341	0.1136	0.0286	0.0318	0.0186	0.0099	0.8834	0.9960
10	−92	3.2407	3.1664	0.7450	0.9131	0.0530	0.0565	0.0091	0.0061	0.0950	0.1343	0.0604	0.0339	0.0106	0.0197	1.0975	1.1890
11	−86	4.5377	3.0263	0.6812	0.6232	0.0669	0.0559	0.0120	0.0084	0.1148	0.1111	0.0157	0.0237	0.0173	0.0151	1.4908	1.4761
12	−63	7.6957	5.2954	0.7900	0.9215	0.0795	0.0839	0.0098	0.0078	0.1133	0.1098	0.0300	0.0222	0.0395	0.0108	0.7932	1.8137
13	−52	3.2983	2.3237	0.9511	0.6386	0.0587	0.0567	0.0087	0.0068	0.1168	0.1058	0.0485	0.0232	0.0231	0.0127	1.4648	1.9583
14	−64	6.6554	4.8795	1.2550	1.1480	0.0880	0.0809	0.0144	0.0109	0.1170	0.1079	0.0099	0.0061	0.0292	0.0385	2.4528	2.4301
15	−71	5.8131	2.3452	0.9683	0.7496	0.0826	0.0564	0.0110	0.0078	0.1221	0.1089	0.0070	0.0284	0.0226	0.0081	1.2126	1.2907
16	−65	4.0100	2.9432	0.9355	0.7461	0.0642	0.0641	0.0123	0.0089	0.1154	0.1109	0.0155	0.0306	0.0131	0.0091	1.7498	1.3237
17	−70	5.6049	6.2525	1.1042	1.1011	0.0842	0.0784	0.0136	0.0126	0.1266	0.1072	0.0060	0.0170	0.0300	0.0645	3.2616	2.8630
18	−42	3.5198	5.0150	1.0969	0.8776	0.0642	0.0708	0.0076	0.0089	0.1266	0.1062	0.0214	0.0100	0.0089	0.0106	1.3697	1.8835
19	−53	4.7706	4.3064	0.9184	1.1074	0.0732	0.0632	0.0104	0.0077	0.1182	0.1331	0.0229	0.0328	0.0188	0.0089	1.5304	1.6858

*Note*. The patients Nos. 1–11 and 12–19 were categorised high responders (HR) and low responders (LR), respectively, based on the percent change in EASI at 16 weeks from baseline. The relative values of the eight metabolites were calculated at baseline and 16 weeks by metabolome analysis for the 19 patients. EASI: Eczema Area and Severity Index.

**Table 3:** AUC values of the eight metabolites.

Metabolite	Lactic acid	Alanine	Glyceric acid	Fumaric acid	Nonanoic acid	Ribose	Sorbitol	Ornithine
AUC	0.773	0.886	0.773	0.761	0.898	0.920	0.824	0.795

*Note*. AUC: area under the curve.

**Table 4:** Serum concentration of ribose calculated using quantitative analysis at baseline in the 19 patients.

Patient	Ribose (*µ*M)
1	6.02
2	5.34
3	7.23
4	9.49
5	8.54
6	7.88
7	10.38
8	10.59
9	2.52
10	2.68
11	1.34
12	3.24
13	4.32
14	1.11
15	1.52
16	0.76
17	0.50
18	1.65
19	1.38

## Data Availability

The data presented in this study can be made available by the corresponding author/s upon reasonable request.
